# Co-designing a Physical Activity Service for Refugees and Asylum Seekers Using an Experience-Based Co-design Framework

**DOI:** 10.1007/s10903-024-01587-5

**Published:** 2024-04-12

**Authors:** Grace McKeon, Jackie Curtis, Reza Rostami, Monika Sroba, Anna Farello, Rachel Morell, Zachary Steel, Mark Harris, Derrick Silove, Belinda Parmenter, Evan Matthews, Juliana Jamaluddin, Simon Rosenbaum

**Affiliations:** 1 Discipline of Psychiatry and Mental Health, School of Clinical Medicine, University of New South Wales, Sydney, Australia; 2 School of Population Health, University of New South Wales, Sydney, Australia; 3 Mindgardens Neuroscience Network, Sydney, NSW Australia; 4 Loughborough University, London, England; 5 St. John of God Health Care North Richmond Hospital, North Richmond, NSW Australia; 6 Centre for Primary Health Care and Equity, University of New South Wales, Sydney, Australia; 7 School of Health Sciences, University of New South Wales, Sydney, Australia; 8 Centre for Health Behaviour Research South East Technological University, Waterford, Ireland; 9 South Western Sydney Local Health District, New South Wales, Australia

**Keywords:** Physical activity, Co-design, Exercise, Refugee, Asylum seeker

## Abstract

People from refugee and asylum seeker backgrounds resettling in Australia often experience intersecting risks for poor mental and physical health. Physical activity can promote better health outcomes, however there are limited programs tailored for this population. Therefore, understanding how to support refugees and asylum seekers to engage in physical activity is crucial. This paper aims to describe how the experience-based co-design (EBCD) process was used to identify priorities for a new physical activity service for refugees and asylum seekers. Using an EBCD framework we conducted qualitative interviews and co-design workshops with service users (refugees and asylum seekers living in the community) and service providers at a community Centre in Sydney, Australia. Sixteen participants, including eight service users and eight service providers engaged in the EBCD process over 12-months. The interviews revealed common themes or ‘touchpoints’ including barriers and enablers to physical activity participation such as access, safety and competing stressors. Subsequent co-design focus groups resulted in the establishment of five fundamental priorities and actionable strategies; ensuring cultural and psychological safety, promoting accessibility, facilitating support to access basic needs, enhancing physical activity literacy and fostering social connection. Using EBCD methodology, this study used the insights and lived experiences of both service users and providers to co-design a physical activity service for refugees and asylum seekers which is safe, supportive, social and accessible. The results of the implementation and evaluation of the program are ongoing.

## Introduction

Globally, the number of people forcibly displaced due to persecution, conflict, or generalized violence is at an unprecedented level having exceeded 100 million [1]. In Australia, approximately 800,000 refugees and asylum seekers have resettled since 1945 [2]. This population often face difficulties accessing health care and experience poor health outcomes [3]. Addressing the health needs and improving access to health care for this group is critical for protecting fundamental human rights [4].

People from refugee and asylum-seeking backgrounds have complex and intersecting risks for poor mental and physical health due to their past and current migration experiences [5, 6]. This includes high rates of pre-migratory trauma exposure and trauma experienced during the migration experience itself, such as violence, conflict, forced displacement, exploitation and in some cases lengthy periods living in detention or crowded camps [7, 8]. For some, poverty, unsanitary and crowded living conditions, inadequate nutrition and poor access to healthcare services before coming to Australia may exacerbate these risk factors [9].

Exposure to traumatic experiences and extreme stress can continue during and after migration [10]. For example, living stressors such as immigration detention, indeterminate visa status, social isolation and separation from loved ones can contribute to poor mental and physical health [9, 11]. Despite increased health needs, people from refugee and asylum seeker backgrounds often also experience challenges to accessing health services due to cultural, language and financial barriers, compounded by lack of availability, varied access entitlements and supports [12].

Subsequently, prevalence rates of posttraumatic stress disorder (PTSD) are estimated to be greater than 30% in refugee and asylum seekers, compared to a 3.8% lifetime prevalence in the general population [13]. Refugees are also more likely to experience chronic pain [14], rapid weight gain and metabolic disorders such as diabetes [15] compared to people in their host country. There is therefore a critical need to understand and support the health and well-being of refugees and asylum seekers in Australia.

### Role of Physical Activity

There is extensive research showing that physical activity can improve mental and physical health outcomes in the general population [16]. Among refugees and asylum seekers, there is growing evidence to show that sport and physical activity can contribute to psychosocial support [17, 18]. There has also been research showing that leisure time physical activity can improve community cohesion, social capital and wellbeing which are important for resettlement [19]. However, evidence shows that people from refugee and asylum seeker backgrounds engage in low levels of physical activity and are often excluded from physical activity promotion interventions [20, 21]. Therefore, creating targeted health promotion interventions is important to protect the mental and physical health of people from refugee and asylum seeker backgrounds, living in Australia. While we have some understanding of the barriers to participation [22], there are a number of gaps in the literature including actionable strategies to promoting physical activity.

To increase physical activity participation, health promotion efforts need to acknowledge the marginalizing circumstances facing refugees including racism, poverty, domestic and structural violence and trauma, all of which impact physical activity participation and access to programs among refugees and asylum seekers. Co-design is a values-led process centered around five key principles: equal partnership from the beginning; openness to working together towards a shared goal; respect for different views, experiences and diversity; and working together through all stages of the project [23]. Using co-design and co-production to understand how to support people to be physically active, and ensure programs are tailored, inclusive, safe and culturally sensitive, in-depth participatory action research is needed [24]. Such strategies to promote physical activity among marginalized groups, including refugees, is increasing in popularity [25–27]. Therefore, using co-design methodology where lived experience and experiential knowledge of all stakeholders including service users and providers is included and valued, may help to build on the existing knowledge base and create feasible solutions to help overcome these barriers.

Using an experience-based co-design (EBCD) framework [28], this research aims to co-design a new physical activity service for people from refugee and asylum seeker backgrounds living in Sydney, Australia. The approach draws on participatory action research, user-centered design, learning theory, and narrative-based approaches to explore service user and service provider experiences with physical activity and identify priority areas for exercise promotion. This type of participatory action research is increasingly used to inform health care design, and has been shown to improve service user and staff experiences [29].

## Methods

### Design

**Fig. 1:**
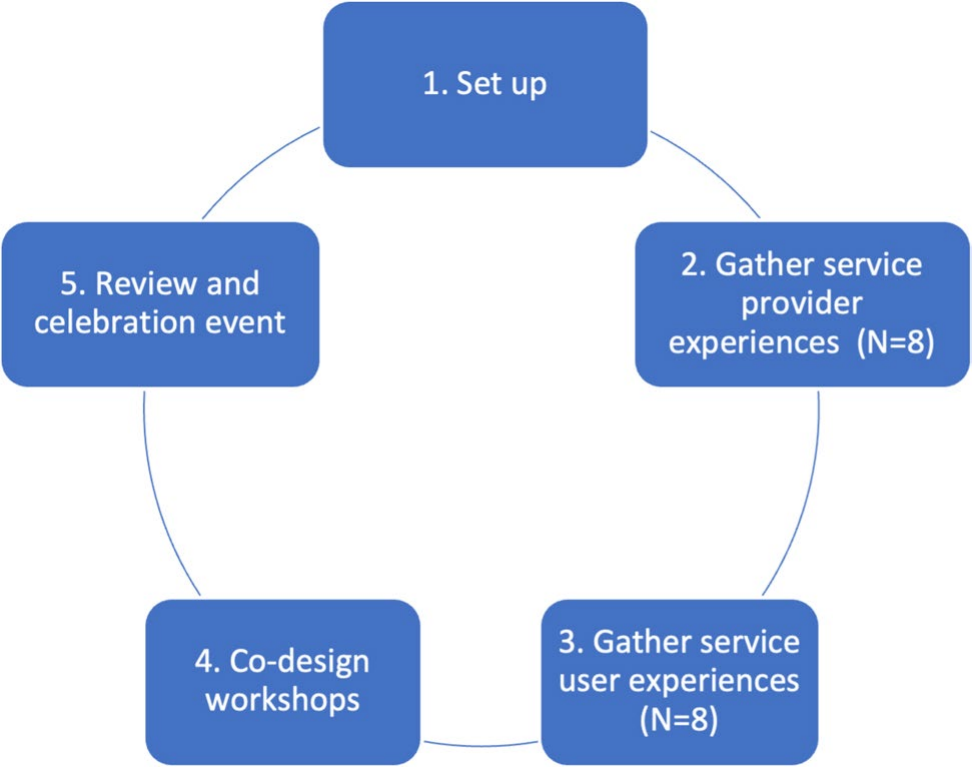
EBCD stages

This study used an Experience Based Co-Design (EBCD) framework, as detailed in Fig. 1 [28]. Primarily aligned with the Australian Healthcare and Hospitals Association (AHHA) EBCD toolkit [30] and the Point of Care Foundation toolkit [31], this study consisted of five stages undertaken over a 12-month period between January and December 2022. This paper describes the EBCD approach, processes and outcomes achieved within the project as a means of sharing and for potential replication by others. It is aligned with SQUIRE 2.0 reporting standards and recommendations for reporting on EBCD studies [32]. Ethical approval was obtained from the UNSW Human Research Ethics Committee (May 2022 / HC220155), prior to recruitment.

### Setting

The project was conducted at Community Centre in Sydney, Australia. The Community Centre provides services to people experiencing social disadvantage including people from refugee and asylum seeker backgrounds, people on low income and migrant communities. Services include a food pantry, legal aid, wellbeing and welfare support.

#### Stage 1—Project development

Key stakeholders were engaged through the establishment of a project steering committee, who subsequently developed the project plan. This committee included research project staff, health professionals working with refugees and asylum seekers and people with lived experience of being a refugee.

#### Stages 2 and 3—Gathering Experiences

##### Participants

The experiences of both service users (people from refugee and asylum seeker backgrounds) and service providers were sought. Snowball sampling was used to recruit eligible participants through a community Centre in Sydney, Australia. Participants were informed about the study through the community Centre via email and word of mouth.

Eligibility inclusion criteria for service users included people over the age of 18 years from a refugee and asylum seeker background and living in Sydney. Eligibility for service providers included people over the age of 18 year providing a health or psychosocial service to refugees and asylum seekers e.g., legal, welfare or mental health support.

##### Procedure

Data were collected through interviews with service providers and service users, representing Stage 2 and 3 of the EBCD framework (Fig. 1). These interviews were conducted by authors GM and SR at a Community Centre. The interviews were semi-structured and included open ended questions about participant experience with physical activity including barriers and facilitators. Interviews were conducted one-on-one, unless a group format was preferred. The questions were modified for service providers, with a focus on their experiences working with refugees and asylum seeker groups, and the barriers and facilitators to promoting physical activity. A bi-cultural interpreter was used during three service-user interviews. Participants were reimbursed for their time with a gift voucher. All interviews were audio and video recorded and transcribed verbatim by the research team.

##### Data Analysis

The interviews were transcribed and uploaded to Nvivo12. Following Braun and Clarkes [33] steps of reflexive thematic analysis, firstly two researchers (GM and MS) became familiar with the interviews after multiple readings of the transcripts. This included note taking to help facilitate immersion in data. Secondly, GM and MS independently coded each interview. An inductive thematic analysis was used to identify new ‘candidate’ themes by combining similar codes to create major categories using a thematic map [33]. The themes, referred to as key ‘touchpoints’ in the EBCD framework [34] were then reviewed, checked against the data, and ‘candidate’ theme names were provided, clearly reflecting the meaning of each. Quotes were anonymised and presented to illustrate the core meaning of themes.

The video recordings were then edited to produce one composite 25-min trigger film, representing all the key touch points. The film showed the key themes generated and experience shaping moments through excerpts of the recorded interviews in a sequenced narrative. The film was created using Adobe Premiere Pro Version 12 and subtitles in English and Dari were included.

#### Stage 4: Understanding the Experience (Co-design)

**Fig. 2:**
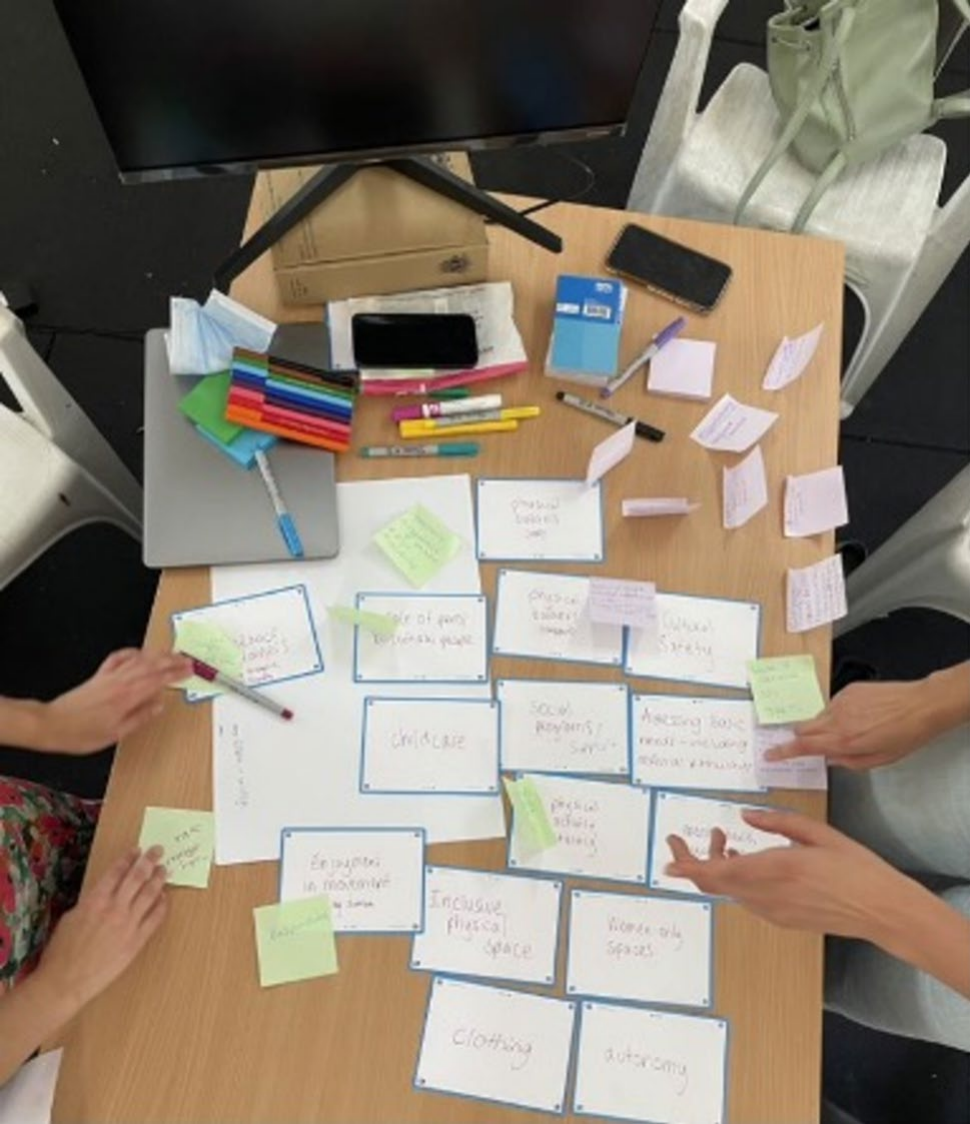
Co-design workshop task where participants wrote down their priorities and ordered them as a group

The co-design phase involved a series of workshops representing Stage 4 of the EBCD framework (Fig. 1). Service provider and service user focus groups were held separately. Co-design workshops were 1–1.5 h in duration and conducted at the Community Centre or via Zoom depending on participant availability. A bi-cultural interpreter was present during two focus groups as needed. Each co-design session was framed by aims and objectives that were outlined at the start of each session. One researcher led the session (GM), while a second researcher observed the co-design work and took field notes (MS). Firstly, the facilitator led the introductions between participants. The trigger film was then played at the beginning of the workshop to present and clarify touchpoints with participants to ensure they solely reflect the experiences shared by the participants, rather than the authors’ perceptions. English and Dari subtitles were provided. Following the film, the facilitator enabled discussion of the film and emerging issues. Following group exercises, participants identified their shared priorities for the new service design and response actions to be implemented (Fig. 2). During co-design workshops the facilitator worked to maintain equal power relations and ensure that all perspectives were considered. In person sessions took place in a circular group format and the facilitator allocated speaking time for all individuals. Co-design workshops were recorded with consent.

#### Stage 5: Review of Priorities

Service providers and service users were consulted for a final review of the priorities identified in Stage 4. This involved distribution via email of the priorities and action points, and an implementation plan for feedback.

## Results

### Participant Characteristics

Sixteen participants including n = 8 service users and n = 8 service providers took part in the co-design process. Most service users were female (75%), from Afghanistan (65%) or Iran (25%). The mean age of service users was 28 years (SD ± 11), ranging from 21 to 52 years. The majority (75%) had been in Australia less than one year at the time of the interview, with the range between 6 months and 10 years (Table 1).

**Table 1:** Service user characteristics—Stages 2 and 3

Variables,	Service users (n = 8) n(%)
*Age range (years)*	
18–30	6 (75)
31–40	19 (12)
41–50	0 (0)
51 +	1 (12)
*Sex, female*	6 (75)
*Country of origin*	
Iran	2 (25)
Afghanistan	6 (75)
*Time in Australia*	
< 1 year	6 (75)
1–3 years	0 (0)
4–10 years	1 (12)
10 + years	1 (12)

Most service providers were female (88%) and 50% of service providers were in executive positions in a psychosocial service, as shown in Table 2.

**Table 2:** Service provider characteristics—Stages 2 and 3

Variables	Service providers (n = 8)
*Age range, yr, n(%)*	
18–30	1 (12)
31–50	5 (63)
51 +	2 (25)
*Sex, female, n(%)*	7 (88)
*Profession*	
Executive	4 (50)
Health professional	2 (25)
Legal	1 (12)
Food	1 (12)

### Stages 2 and 3

**Fig. 3:**
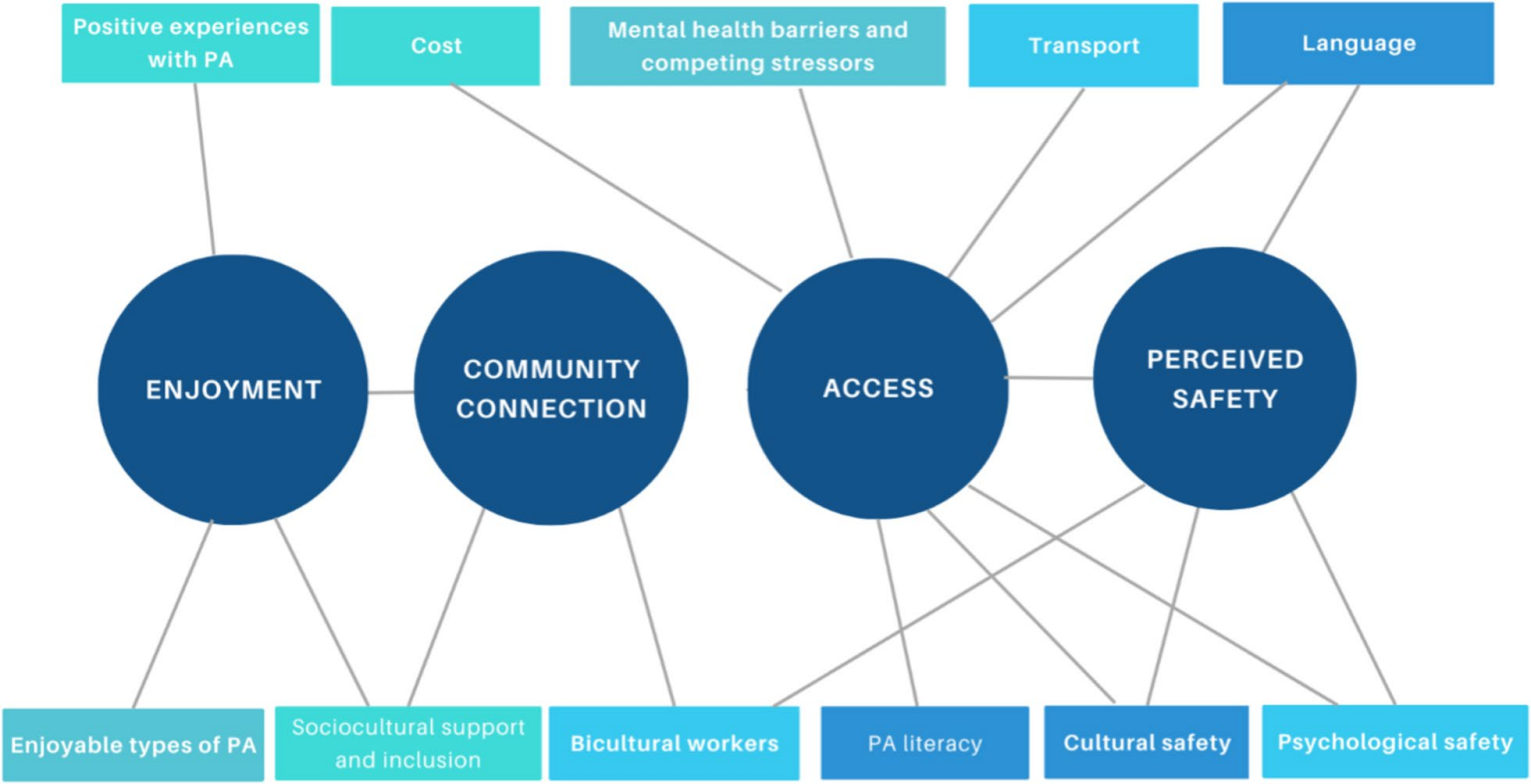
Thematic map of intersecting themes and subthemes

Thirteen participants took part in one-on-one interviews with the interviewer, while three participants opted for a group interview format. Four key themes/“touchpoints” and 11 subthemes were generated from service user and provider interviews. Figure 3 details a thematic map of the overlapping and intersecting themes and subthemes. The touchpoints were similar between service users and providers and have been combined with exemplar quotes in Table 3. The final video included all touchpoints identified by the service-users and providers.

**Table 3:** Themes and exemplar quotes

Theme	Subtheme	Exemplar quotes
Difficulties with access	Mental health barriers and competing stressors	“It’s becoming 8 months that I didn't do any exercise because of some problems, some mental health problems and depression and so many other problems in here”—**Service user- P3** **“**I say since early, the before government granted my visa, I didn’t [do] any exercise and physical activity because the all the time I experience anxiety and depression and all that I’m experiencing PTSD and backlash to all negative attitude for my home country and the journey or something like that**” Service user- P16** “ So basically, the main issue has been number one access obviously, then financial difficulty hardship main one, mental health issues related to families being overseas, their settlement issues where they have had to learn how to figure out how to be settled integrated this society, but also housing has been an issue, just getting Centrelink benefits, working things out…………So gym really has been the last thing on their mind, sports and all that.”—**Service users- P5,6,7** “ This is the difference the attitude to member of family negative affected to doing to physical activity and exercise because we cannot manage based on money, based on pocket, based on job, based on time.” **Service user- P6** “ This time my priority is living certainty, no physical activity. Exactly physical activity a positive impact to mine, but this is not my priority. My priority is that get money for support my family, support my daughters, support my wife”—**Service user – P16** “ Maybe bring some [food hamper] boxes, or maybe some exercise clothing and you have someone who’s taking care of the kids. And it’s a one stop, they picking up the kids and they are doing 35 min of exercise and then they go out and they already have their food”—**Service provider** – **P8** **“** I think creating a space that is welcoming, that people feel that they belong to and are able to come when they need support, where they are able to access additional supports, where they able to be connected to other different opportunities to build their fitness goals, their health.”—**Service provider- P15** “So I think we need to partner up. We need to say, we need to offer a service that it’s a bit holistic in that way, that we say we understand that you have this basic needs and this is a place where I can help you access those basic needs.” **Service provider** – **P8** **“** I think you should start thinking of having like a little someone taking care of the kids for 45 min”—**Service provider-P8**
	Cost	“ We can’t go because we don't have car and also lack of income. Because here in Australia we are new, we don’t have any job and we have to pay for the gym, and that’s the point, our barriers to the gym”—**Service User -P3** “ They can’t afford it. This in money from that little Centrelink benefits that they get they had to fend for themselves and to pay for their rent and as well as support for families overseas.”**: Service User**—**P5,6,7** **“**100% finance. If you’ve got children, you’re not going to spend [money on exercise]”- **Service Provider – P9** “I think as well just obviously the cost factor is always one of the biggest prohibiting factors, so by being that free space that’s that’s very valuable”** Service Provider- P15** “There is beauty of charging people something. Because it is stop from being charity and something that I have in push to something that I have agency with…… it empowers you to think that you belong. So it could be a dollar donation. It also means that that money when you get it and you don’t have to spend it, you can say, ‘ hey what do you think we need? What would you like to do ?’ Like this is make them feel part of it.” **Service Provider- P8**
	Transport	“ We live very far from here. Well I know you you guys can’t get the transport to bring them from there. But at least if we can organise some transportation bus or something from train station to here it’s going to be great.” **Service User- P2** “ I really prefer about a transport and some, like some way to help them to bring them here. Because some of them don’t know the way and some of the girls which come new to Australia they cannot speak English, so it’s much powerful for them”—**Service User- P2**
	Language barriers	“ Yeah because for Asylum seeker and refugee there is more barriers. For example if came in Australia the language barriers. This is the big problem. Sometimes I think scared or going to gym or sports like I don’t talk to someone, if they talk to me I cannot respond to them and explain my situation.”—**Service User- P16** “They can’t communicate. It’s the worst thing. That’s the number one barrier to mental health and everything, everything else, you know.” **Service User- P5,6,7** **“**And it’s good to some, one person be with them, even if like suppose, if you if you will be there you say, ‘I’m interpreter for everyone that they don’t know English’. They couldn’t understand English, we can play something that around her language or maybe we can Google it. Or we will find some way for them to they should understand.”**—Service User—P2**
	PA literacy	“As all we know activity is so important for health, for mental health, for physical health, for like reduce lots of umm disease? like cancer, like this. So good for the health.”—**Service User- P3** “She said she attended gym for last 6 months previous to coming to Australia. She believes that it helped her physical as she had some migraines, severe migraines and headaches. And she knows, she correlates that to going to gym and it helped her get better. So she now knows that it helps some, certain health issues, physical health issues. So it actually reduced her headaches which has actually increased when, since she’s come to Australia, cause she is not active at all.” **Service User- P7** “ Everything was normal I got to the gym. But when Taliban came they even, they want they just closed the universities and schools. Probably they close, 100% they going to close the gyms as well. Straight away they closed the gym for the ladies and then everyone scared of going to outside. How they can go to the gym and do the physical activities? Everyone was stuck at home with stress, with lots of lots of them had a problem. Like I had a problem, doctor says you have to go to the gym, this is your medicine. And then when I lost it, I had my problem back because of that. And then everyone’s there is no physical activity now.” **Service User- P2** “ So exercise is important. There’s a consciousness of how important it is. People are told that they have to do it but they don’t have the access to doing it.”—**Service Provider- P9** “ Health literacy, very low yeah. So very low health literacy, sort of navigating the system as well. Like how to get services, how to access services, things that are free, things that are not free, those kinds of things. ……… Like even though I say health literacy is an issue, they are aware of the things they don’t know….Like as in there like we want to, but there are so many barriers that it’s not worth doing it.”—**Service Provider-P13** **“** Because I am not good at workout properly, I need some ideas, what is the best way. So imagine after 8 years in detention, sometimes I feel I am very tired, like my physical body, I get a lot of pain.” **Service User -P1** “Look I’ve never been to the gym before. I don’t know what’s the good tools or what the good machine to bring.” **Service Provider- P10** “ Like the positive of peer to peer is that you have that cultural awareness, but whether people will actually take it seriously enough to follow the instructions etc. Versus getting it from someone who’s like qualified instructor.”—**Service Provider- P13** “ I think peer is good. But honestly and I’m telling you this from 30 years of experience, that off.., that in many of these cultures it is about the experts. ….. there is not a culture that doesn’t respect a doctor.”—**Service Provider- P9**
I need Perceived safety	Cultural	“The most important thing is that the time like women and men will be separate like because girls can’t like doing exercise with men as well, because we are again girls and Muslims as you know.” **Service User- P3** “ And it makes us very feel like very motivated, that we going to have to place that it’s only for the girls and we can would feel free, we can come and exercise over here. Without that I just found 2–3 gyms around my, close to my home but it was not only for the ladies. I think it’s very less to find that gyms for only, for the girls. That’s why I got nervous that, where should I go? what should I do ? I didn’t know it about these places in Australia.” **Service User-P2** “ How to make exercise be a part of their cultural experience. They love walking. They love dancing. They love gardening. They like tasks that they can see, you know has some outcome at the end” – **Service Provider- P11** “In Afghanistan they had to go to a women’s gym. Here they don’t mind a mixed gym, they would prefer a mixed gym.”—**Service Users- P5,6,7** “I think something again that is very culturally appropriate, because a lot of women, Muslim women, you know, women from sort of which is like a lot of the refugees called tend to want areas that are women only, tend to want curtains if possible. Especially if they’re going to be working out where you know it, I look,it’s that thing whether someone more conservative than others. Obviously I’m not going to brush everyone with the one stroke. So there are people who are like, “ no it’s fine I’m happy with doing whatever”, but then the more conservative ones which are the ones you actually want to target because they’re the ones we don’t do anything and they are the ones who are going to be more disadvantaged.”—**Service User- P5,6,7** “ The exercise physiologist that you have, it would be really good ….. for them to do some cultural training, cultural awareness training to make them aware, more aware of the people that are going to be in front of them and some of the issues that may come up and how you resolve some of those issues”—**Service Provider- P11** “physical appearance matters so much, but not in the common in like is she pretty or not. It is in the way you make people feel comfortable.”—**Service Provider-P8**
	Psychological	“ And of course there are challenges, post traumatic stress, a lot of them have been tortured. All of that. But asylum seekers some of them have been waiting for permanent residency for more than eight years. They’ve been in detention and the trauma that comes out in unexpected ways when you talk to them and when you deal with them. It, you need to understand their circumstances to understand why they you know react the way they do and think.” – **Service Provider- P11**
Community Connection	Sociocultural support and inclusion	“Look one thing I like it here in the community centre, when you go in centre and if you know all the people here working, if you like you go in your big family.” **Service Provider- P10** “ So having that resource for them to be able to wear a uniform, a uniform that they would come and say ‘oh this is us, we are coming, we are part of this little research gym’, you know.”—**Service Provider- P14** “ But when I am in a group of good people, I feel I can exercise a few times better. I can see the vibe. I can see the happiness. When someone shares smiles, when someone is telling me about how they changed their lives through exercise and through a lot of activities, about their life when they share their stories, then I can share my story and I think this is the way we get connected with each other”—**Service User- P1** **“** So to get people here early on, to allow them to integrate into a society through physical activity and movement is huge.”—**Service Provider- P11** “The first step refugee and asylum seeker need good connected, connection to home community in host country. For example, the Iranian, Afghan, Syrian, Arab, Lebanese these all or something like that”—**Service User- P16** “ What I think is that, to see different circles of society, migrants, Aboriginal people, refugees, anyone who had difficult lives before. Even people from umm, like people who were born in Australia. Anyone together, to share hope together and be like a family. And if someone is not okay we can help them to be okay. We can be together and as I said when we share hope together that is the time, that is the point.”—**Service User -P1** “An Australian community adjustment because if this community adjustment this is the big processing. You’re the first step you have to feeling confident and then go to community and there feeling positive and improvement your talent or capacity. “-**Service User—P16** “So I don’t mind what is her culture what’s her background, what’s her past I don’t care about that. My speech is always racism is not welcome”—**Service User -P4**
	Bi-cultural workers	“ Like you meet someone in the community, they make a referral, they come with them, they sit with them the first time. It’s a trust building exercise and you survey each phase of that.”—**Service Provider- P8** “ The best way is that all education is institute have to writing the best program and they delivered this program to local area and the local area and the local government, for example the council, deliver this knowledge to community leader and community leader transfer to member of community.”—**Service User: P16** “ We are, we want to promote it within the communities. So finding community connectors or possibly training these girls to become the community connectors and possibly pay them”—**Service Provider- P14**
Physical activity should be enjoyable	Positive PA experiences	“ Personally when I did exercise I feel more energy. I, all my tiredness from my body go out and I receive more energy and I become so hopeful in my life and full of energy and like strong feeling and it’s really good for mental health.” **Service User- P4** **“** When I got free (from detention), I had, I was very weak. Imagine for 15 months I was locked up in a room. And umm, I had a personal trainer for a couple of months. He helped me a lot and I also did yoga sessions for 3 months. I couldn't move my hands when I got free, like I couldn't bend, couldn't touch the ground, touch the floor. But After a few months I felt that I can do that.”- **Service User-P1** “ I have experience after gym and after exercise I feel so relaxed. Like so good, full of energy, and we can make lots of decision when we feel relaxed. And also our sleep quality will become good”- **Service User- P3** **“**They are so interested to join a gym because they need not just for body and not just for health, but it’s so good for their mental health. Yeh, because we are all in depression you know and anxiety, we are all far away from family. And we need to do exercise, because it will be lots of help for our mental health. **Service User- P3** “Another my opinion about football team is my equality.….They will never judge me from where I am and what’s my language. But we all care about one ball and we run for one ball and for one goal.”- **Service User- P4**
	Enjoyable types of PA	**“**I love to to attend in a gym which there is a freedom and everybody do whatever they like. So we should have like a different stuff, which is music, so if I love to dance so I can dance in the corner, so if I like to use a machine, if I if I love to use a free sports”—**Service User- P4** “ You can help us first of all make a schedule for us. And when we come here um like we should doing lots of exercise, like fun together, have fun together. “- **Service User- P3** “ So a freedom hall which looks like like give everybody their rights. I would love to join that kind of gym.”—**Service User- P4** “ You know what gym is not possible without music. We have to have the music for the Zumba. Zumba is good.”—**Service User- P2** “ At least we need to make a room for people who still tired about life and they can come here and change their mind and go back happy for the home.”—**Service User- P4** **“**A lot of humour, laughter, music, food and the social aspect.—**Service Provider- P11**

### Stages 4 and 5; Agreed Priorities

Focus groups were conducted with N = 14 participants. One participant withdrew from the research and a second was overseas at the time of the co-design workshops. The key priorities identified through the EBCD process centered around creating a safe, inclusive and accessible service that acknowledges and respects people’s culture, experience and identify. Additionally, providing support to address basic needs beyond the physical activity service, improving physical activity literacy among the participants, providers and community leaders, and creating social and community connections are important components. A summary of the key priorities and response action points are shown in Table 4.

**Table 4:** Key priorities and action points

Priorities for EBCD	Co-designed response action points
Cultural safety *To create a service where identity, culture and experience are acknowledged, welcomed and respected*	Staff require cultural competency training
	Ensure staff are respectful, compassionate, friendly and understanding
	Provide options for women only times and spaces
	Provide privacy including curtains on windows and spaces to get changed
	Engage with bi-cultural workers/community connectors to build trust, assist with referrals and link between communities and program
	Respond to language barriers by engaging bilingual speakers to facilitate sessions for people from non-English speaking backgrounds
Emotional safety *To create a service where people feel understood and supported*	Establish partnerships and strong referral pathways to existing mental health services
	Ensure staff have received mental health and trauma informed training and understand the impact of trauma and the refugee experience
	Use safe and nurturing features and environment including colors, art, music
	Establish trust and transparency with the community and referrers by consulting with them on how they want to be involved e.g., steering committees
Accessible *To identify and address barriers to accessing the service e.g., cost, location*	Free or reduced cost to access the service
	Outreach/co-location for those who cannot physically access or transport options from local train station
	Improve access for women and carers by providing childcare options e.g., creche, children and mother classes, linking class times for mothers with childcare services
Support to address basic needs *To provide additional support beyond the exercise program which helps people to meet their basic needs e.g., housing, food*	Partner with existing services and establish strong referral schemes to facilitate referrals to other appropriate services
	Translated written materials with information about other services
	Co-located with other services e.g., food pantry, legal, welfare aid
Physical activity literacy *To improve physical activity literacy among refugee and asylum seekers and community leaders*	Employ university qualified professionals to facilitate the exercise program e.g. accredited exercise physiologists who can: Tailor exercises to individual complex needs Make the service credible for referrals into the program
	Focus on community development- e.g., upskilling community leaders on the benefits of physical activity and how to support others to be active so they can become leaders in physical activity promotion
	Engage staff and potential referrers in the exercise program
Social and community connection *To create a community-based program which provides people with a sense of connectedness*	Led by the community and designed by people with lived experience
	Provide group-based programs with people from different cultural backgrounds to create social opportunities
	Offer physical activity modalities that are social and fun e.g., dance/Zumba, strength, yoga, sport
	Consider soft entry through activities which have a tangible outcome and may be more culturally appropriate- e.g., gardening, art

## Reflexivity

The interviews and focus groups for this study were conducted by GM and SR. GM is a female researcher with previous experience in qualitative research related to physical activity and mental health. GM acknowledges the potential for her own cultural and social biases to influence the research process, given her Anglo-Celtic background. GM led all the interviews with female participants. SR is a male researcher with previous experience in qualitative research focusing on physical activity, mental health and displaced communities. Strategies to address potential power imbalances included the involvement of a bicultural worker/interpreter when preferred by participants, and feedback from the broader research committee throughout, which includes individuals with diverse professional, cultural and religious backgrounds. RR, member of the research committee and co-author has lived experienced as a refugee and provided insight throughout the process. We also presented the findings back to participants mid-way through the process via a short film and again via email at the end of the analysis to ensure participants perspectives were accurately reflected.

## Discussion

Using EBCD methodology, this study fills a gap in the literature by utilising the insights and lived experiences of both service users and providers to co-design a physical activity service for refugees and asylum seekers. The first stage of the EBCD process identified touchpoints that focused on enablers and barriers to physical activity participation including access (e.g., finances, transport), safety (e.g., lack of culturally safe spaces to be active) and competing stressors (e.g., employment difficulties). While these barriers were already understood [35], jointly, service users and providers identified five fundamental priorities with actionable strategies to addressing them: ensuring cultural and psychological safety, promoting accessibility, facilitating support to access basic needs, enhancing physical activity literacy and fostering social connection.

### Emotional and Cultural Safety

The EBCD process identified fostering safety as the most important priority for the physical activity service. Safety involves protecting individuals from emotional, physical and psychological harm while participating in physical activity. Actionable strategies focused on workforce capacity building including upskilling staff running the service in cultural safety and trauma informed practice. Participants also discussed the importance of engaging with bicultural workers and community connectors to build trust, facilitate referrals and co-facilitate sessions for people from non-English speaking backgrounds.

Key strategies acknowledged by service users and providers to create a service that is safe for people from different cultural and religious backgrounds include establishing women only times and spaces, privacy, and culturally capable staff. Previous research among culturally and linguistically diverse groups in Australia has found similar ethnic specific barriers including cultural modesty in the form of appropriate dress and privacy as a barrier for women’s participation [36]. This research suggested having women only classes, maintaining closed of sections of the gym facility and empowering women by placing them at the center of program development and encouraging them to take lead roles in programs [36].

Overlooking culture in healthcare has been identified as one of the most pertinent barriers to advancing the highest level of health care worldwide [37]. Previous studies have shown that cultural safety training can improve healthcare providers ability to understand and address the cultural and linguistic needs of refugees and improve health outcomes [38]. Yet, existing cultural competency frameworks in Australia for clinicians exclude exercise professionals [39], despite the critical role they play in health promotion, or focus solely on Aboriginal and Torres Strait Islander cultural competency [40]. In addition to improving health outcomes, making participants feel safe to express their identities can help individuals acknowledge and appreciate each other’s cultural values [41]. Strategies to create safe environments include having ‘cultural insiders’ as staff members within organisations and allowing people the opportunity to reconnect and develop their ethnic identifies [42].

Ensuring emotional and psychological safety was also identified as a priority in the current research. Therefore, consideration of trauma in the design and delivery of the service was recommended. Acknowledgement of the importance of trauma informed practice and the implementation into physical activity programs has been increasing in popularity [43]. During the co-design workshops, participants recommended that staff leading the physical activity sessions receive mental health training. Service users expressed a desire for staff members to understand and accommodate their mental health needs, and not to add additional pressure around attendance or performance if they were lacking motivation or experiencing distress. Among refugees, previous research has focused on creating trauma informed sports programs as opposed to physical activity [44]. Previous research has suggested that focusing on and promoting personal progress rather than performance, being available for informal time before or after sessions, inviting players to provide input to make the experience better can all be applied in contexts, such as that of the current research.

### Accessibility

The EBCD process also revealed that the physical activity service needs to be easily accessible. Service users reported cost as a major barrier to participation since being in Australia and identified that the service would need to be little to no cost. This finding aligns with research on leisure time activity among refugees in Australia which showed that cost was identified as the highest barrier to physical activity, and has a significant relationship with reduced levels of participation [45].

Transport was also raised as a barrier and it was identified that many refugees and asylum seekers live approximately one hour from the community Centre via public transport and therefore would have difficulties accessing the service. Suggestions included outreach services to reach these priority populations or transport options, for example picking people up from the local train station was among the recommendations from the co-design participants. The timings of sessions also need to be flexible to accommodate changes in family and employment situations. Although service users did not report childcare responsibilities as a barrier, possibly due to many not having children, providers emphasised the significance of offering mother–child class options. This is important, given previous research among a group of Muslim women in Australia aligned with the service providers view, whereby women often perceived physical activity participation as conflicting with their ‘ethic of care’ towards family responsibilities, potentially leading to neglect of their role [36]. Therefore, the service must address geographical, linguistic, family and financial barrier to improve access for this population.

### Support to Access Basic Needs

While the importance of physical activity as a means of promoting physical and mental well-being was acknowledged and valued by all participants, many reported having competing stressors that made it challenging to participate. Stressors included navigating the complex resettlement process, employment difficulties and a lack of mental and physical energy. It was identified during the co-design workshops the need to acknowledge the distinct differences in challenges facing those on secure versus temporary visas. Greater support should be considered for people on bridging visas who may be facing these additional stressors, living in limbo with uncertainty about the future [46].

Service users and providers reported that staff providing the physical activity service should be aware of differences in visa status between service users and establish strong referral pathways to facilitate referrals to appropriate services. Additionally, participants recommended translating written materials with information about other services to make them more accessible to refugees with limited English proficiency. Lastly, co-locating physical activity programs with other services such as food pantries, legal services, or welfare aid could facilitate easier access and participation for refugees who are dealing with multiple stressors. These recommendations highlight the importance of taking a holistic and collaborative approach to support the physical activity and well-being of refugees in the face of competing stressors, as anticipated during the services’ establishment. The findings also caution against creating a physical activity program in isolation as a standalone activity, disconnected from an established agencies designed to provide assistance with these complex needs.

### Social and Community Connection

Prioritising social and community connection was identified as a core component of physical activity promotion for refugees and asylum seekers. Service users and providers recommended that the service incorporates group-based activities which are enjoyable and fun. Different modalities were suggested including yoga, Zumba and dance. This finding aligns with research showing that sport can foster integration and can promote sustainable engagement and social inclusion for refugees and participation in enjoyable group activities even in the short term has shown to strengthen bonding connections [47]. Consideration of soft entries to the program through culturally acceptable activities such as painting or gardening were also suggested to help engage people who may be hesitant to join. In addition, given the established association between depressive symptoms and reduced community engagement with one’s own community in their host country [48], supporting those experiencing mental health symptoms to engage may help to foster positive adaption for resettled refugees.

Partnership between the community center, providers and the community were identified in the interviews and focus groups, as critical to the long-term sustainability of a physical activity service. Participants identified that their involvement and those of the community were important in the decision-making process. Therefore, researchers, policy makers and practitioners should engage in continuous dialogue with refugees and asylum seekers throughout the design, implementation and evaluation of physical activity programs, rather than seek a ‘quick’ solution.

### Physical Activity Literacy

A need to target physical activity literacy among refugees and asylum seekers and health service providers and was identified as a priority. It was also identified that there is not a shared understanding of the role of leisure time physical activity among service users but also their communities more broadly. Service users often identified that they were not confident on what exercises to do or how to do them and it was agreed that employing university qualified exercise professionals who can tailor exercises was important. The service providers suggested that this would also improve the credibility of the service and subsequently boost referrals from other health or psychosocial services.

It was also recommended that the physical activity service engages with community leaders and service providers to upskill them on the benefits of physical activity. These educators can play a critical role in promoting and role-modelling physical activity and it has been shown to improve the effectiveness of other chronic disease prevention efforts [49].

### Reflections and Limitations

Using the EBCD methodology has been both challenging and rewarding. Barriers we faced included a lack of trust of the researchers by some participants due to their association with a university, fluctuating health needs of service users and difficulties with relationships between participants from diverse professional and life backgrounds. We also experienced challenges and lengthy delays scheduling focus groups, especially when a interpreter was needed, or participants were not confident with transport. Similar challenges have been experienced by other researchers adopting the EBCD process and conducting community-based research [29, 50, 51].

Despite these challenges, we are confident that this process involved authentic input and insight from people from refugee and asylum seeker backgrounds. The process has helped to build trust between the researchers, stakeholders and the community and provide a sense of autonomy and ownership in the process. Key learnings have included having flexible timelines and adaptable delivery methods to foster meaningful community engagement. For example, we were required to adapt some focus groups to an online format and/or have hybrid options. We have also learnt to be open to criticism and accountability at every stage of the process.

Importantly, this work has not only identified barriers to physical activity, but strengths in an underserved community and allowed solutions to be created using the combined strengths of lived experience with research methodology. We therefore argue for the use of participatory approaches such as EBCD methodology with people from refugee and asylum seeker backgrounds to develop more culturally relevant, gendered, and local understandings and to utilise these insights within program co-design and delivery.

It is however important to acknowledge there were limitations, given the co-design is tailored to one service at a single site in NSW, Australia. The sample size is small, the majority of participants were female, and the transferability of findings is limited given we only interviewed people from one geographic region. The gender differences in our research may be attributed to the fact that women are often disproportionately affected by migration [52], and likely face additional barriers to physical activity participation which may have motivated them to share their experiences and needs. The study was also led by a woman researcher which may have influenced participants sense of comfort in taking part.

## Conclusion

EBCD provides a novel framework for engaging service users and providers and this study reflects on the outcomes and process. The findings from this process provide practical actions for implementing a physical activity service for people from refugee and asylum seeker backgrounds which ensures cultural and psychological safety, promotes accessibility, facilitates support to access basic needs, enhances physical activity literacy and fosters social connection. The resultant physical activity service which aims to be accessible and meet the needs of people from refugee and asylum seeker backgrounds living in Australia is being implemented and the evaluation is ongoing.
